# Supplementary material for: Health improvement of the elderly in five Central Asian countries during COVID-19 based on difference game
Source: PLoS One. 2023 Dec 5;18(12):e0294697. doi: 10.1371/journal.pone.0294697 (PMC10697614; doi:10.1371/journal.pone.0294697)
Supplement: S1 File — (DOCX) [file pone.0294697.s001.docx]

# **Appendix**

**Appendix 1**

Take the derivatives of *M_N_*_1_ and *I_N_*_1_ respectively with respect to (13), and take the derivatives of *F_N_*_2_ with respect to (14), and set them equal to zero, we can get:

$M_{N1}^{\text{*}}\left(t\right)\text{=}\frac{1}{c_{M}}\left(b+\frac{\partial V_{N1}}{\partial x_{N1}}\ln\left(\beta+1\right)\right)$, $I_{N1}^{\text{*}}\left(t\right)\text{=}-\frac{c_{I}}{2\lambda_{1}}\left(\frac{\partial V_{N1}}{\partial x_{N1}}\right)^{-1}$ (41)

$F_{N2}^{\text{*}}\left(t\right)\text{=}\frac{\lambda_{2}+\lambda_{3}-\lambda_{4}}{c_{F}}\left(\frac{\partial V_{N2}}{\partial x_{N2}}\right)$ (42)

Substituting (41) into (13) and substituting (42) into (14), we can get:

$\begin{matrix} \rho V_{N1}=\left[b\frac{1}{c_{M}}\left(b+\frac{\partial V_{N1}}{\partial x_{N1}}\ln\left(\beta+1\right)\right)+c_{I}\frac{c_{I}}{2\lambda_{1}}\left(\frac{\partial V_{N1}}{\partial x_{N1}}\right)^{-1}-\frac{c_{M}}{2}\left(\frac{1}{c_{M}}\right)^{2}\left(b+\frac{\partial V_{N1}}{\partial x_{N1}}\ln\left(\beta+1\right)\right)^{2}\right. \\ \left.+l_{1}x_{N1}\left(t\right)\right]+\frac{\partial V_{N1}}{\partial x_{N1}}\left[-\lambda_{1}\left(\frac{c_{I}}{2\lambda_{1}}\right)^{2}\left(\frac{\partial V_{N1}}{\partial x_{N1}}\right)^{-2}+\ln\left(\beta+1\right)\frac{1}{c_{M}}\left(b+\frac{\partial V_{N1}}{\partial x_{N1}}\ln\left(\beta+1\right)\right)-\delta x_{N1}\left(t\right)\right] \end{matrix}$ (43)

$\rho V_{N2}=\left[-\frac{c_{F}}{2}\left(\frac{\lambda_{2}}{c_{F}}\right)^{2}\left(\frac{\partial V_{N2}}{\partial x_{N2}}\right)^{2}\right.\left.+l_{2}x_{N2}\left(t\right)\right]+\frac{\partial V_{N2}}{\partial x_{N2}}\left[\frac{\left(\lambda_{2}+\lambda_{3}-\lambda_{4}\right)^{2}}{c_{F}}\left(\frac{\partial V_{N2}}{\partial x_{N2}}\right)-\delta x_{N2}\left(t\right)\right]$ (44)

Let $V_{N1}^{*}=m_{1}x_{N1}+m_{2}$,$V_{N2}^{\text{*}}=m_{3}x_{N2}+m_{4}$, wherein, *m*_1_, *m*_2_, *m*_3_ and *m*_4_ are all constants. The parameters of the optimal social welfare function can be obtained by calculation as follows:

$\left\{\begin{matrix} m_{1}=\frac{l_{1}}{\rho+\delta} \\ m_{2}=\frac{1}{\rho}\left[b\frac{1}{c_{M}}\left(b+\frac{l_{1}}{\rho+\delta}\ln\left(\beta+1\right)\right)+c_{I}\frac{c_{I}}{2\lambda_{1}}\left(\frac{l_{1}}{\rho+\delta}\right)^{-1}-\right.\left.\frac{c_{M}}{2}\left(\frac{1}{c_{M}}\right)^{2}\left(b+\frac{l_{1}}{\rho+\delta}\ln\left(\beta+1\right)\right)^{2}\right] \\ +\frac{l_{1}}{\rho+\delta}\frac{1}{\rho}\left[-\lambda_{1}\left(\frac{c_{I}}{2\lambda_{1}}\right)^{2}\left(\frac{l_{1}}{\rho+\delta}\right)^{-2}+\ln\left(\beta+1\right)\frac{1}{c_{M}}\left(b+\frac{l_{1}}{\rho+\delta}\ln\left(\beta+1\right)\right)\right] \end{matrix}\right.$ (45)

$\left\{\begin{matrix} m_{3}=\frac{l_{2}}{\rho+\delta} \\ m_{4}=-\frac{1}{\rho}\frac{c_{F}}{2}\left(\frac{\lambda_{2}+\lambda_{3}-\lambda_{4}}{c_{F}}\right)^{2}\left(\frac{l_{2}}{\rho+\delta}\right)^{2}+\frac{l_{2}}{\rho+\delta}\frac{1}{\rho}\frac{\left(\lambda_{2}+\lambda_{3}-\lambda_{4}\right)^{2}}{c_{F}}\left(\frac{l_{2}}{\rho+\delta}\right) \end{matrix}\right.$ (46)

Therefore, it can be concluded that:

$\begin{matrix} V_{N1}^{*}=\frac{l_{1}}{\rho+\delta}x_{N1}+\frac{1}{\rho}\left[b\frac{1}{c_{M}}\left(b+\frac{l_{1}}{\rho+\delta}\ln\left(\beta+1\right)\right)+c_{I}\frac{c_{I}}{2\lambda_{1}}\left(\frac{l_{1}}{\rho+\delta}\right)^{-1}-\right. \\ \left.\frac{c_{M}}{2}\left(\frac{1}{c_{M}}\right)^{2}\left(b+\frac{l_{1}}{\rho+\delta}\ln\left(\beta+1\right)\right)^{2}\right]+\frac{l_{1}}{\rho+\delta}\frac{1}{\rho} \\ \left[-\lambda_{1}\left(\frac{c_{I}}{2\lambda_{1}}\right)^{2}\left(\frac{l_{1}}{\rho+\delta}\right)^{-2}+\ln\left(\beta+1\right)\frac{1}{c_{M}}\left(b+\frac{l_{1}}{\rho+\delta}\ln\left(\beta+1\right)\right)\right] \end{matrix}$ (47)

$V_{N2}^{\text{*}}=\frac{l_{2}}{\rho+\delta}x_{N2}-\frac{1}{\rho}\frac{c_{F}}{2}\left(\frac{\lambda_{2}+\lambda_{3}-\lambda_{4}}{c_{F}}\right)^{2}\left(\frac{l_{2}}{\rho+\delta}\right)^{2}+\frac{l_{2}}{\rho+\delta}\frac{1}{\rho}\left(\lambda_{2}+\lambda_{3}-\lambda_{4}\right)^{2}\frac{1}{c_{F}}\left(\frac{l_{2}}{\rho+\delta}\right)$ (48)

In this case,

$M_{N1}^{\text{*}}\left(t\right)\text{=}\frac{1}{c_{M}}\left(b+\frac{l_{1}}{\rho+\delta}\ln\left(\beta+1\right)\right)$ ,$I_{N1}^{\text{*}}\left(t\right)\text{=}-\frac{c_{I}}{2\lambda_{1}}\left(\frac{l_{1}}{\rho+\delta}\right)^{-1}$ (49)

$F_{N2}^{\text{*}}\left(t\right)\text{=}\frac{\lambda_{2}+\lambda_{3}-\lambda_{4}}{c_{F}}\frac{l_{2}}{\rho+\delta}$ (50)

**Appendix 2**

Take the derivatives of *M_H_*_1_ and *I_H_*_1_ respectively with respect to (15), and take the derivatives of *F_H_*_2_ with respect to (16), and set them equal to zero, we can get:

$M_{H1}^{\text{*}}\left(t\right)\text{=}\frac{1}{c_{M}+c_{H}}\left[b+\frac{\partial V_{H}}{\partial x_{H}}\ln\left(1+\beta+\beta_{H}\right)\right]$, $I_{H1}^{\text{*}}\left(t\right)\text{=}-\frac{c_{I}-\alpha_{1}}{2\lambda_{1}}\left(\frac{\partial V_{H1}}{\partial x_{H1}}\right)^{-1}$ (51)

$F_{H2}^{\text{*}}\left(t\right)\text{=}\frac{\lambda_{2}+\lambda_{H}}{c_{F}+c_{H}}\frac{\partial V_{H2}}{\partial x_{H2}}$ (52)

Substituting (51) into (15) and substituting (52) into (16), we can get:

$\begin{matrix} \rho V_{H1}=\left[b\frac{1}{c_{M}+c_{H}}\left[b+\frac{\partial V_{H}}{\partial x_{H}}\ln\left(1+\beta+\beta_{H}\right)\right]+\frac{\left(c_{I}-\alpha_{1}\right)^{2}}{2\lambda_{1}}\left(\frac{\partial V_{H1}}{\partial x_{H1}}\right)^{-1}\right. \\ \left.-\frac{1}{2}\frac{1}{c_{M}+c_{H}}\left[b+\frac{\partial V_{H}}{\partial x_{H}}\ln\left(1+\beta+\beta_{H}\right)\right]^{2}+l_{1}x_{H1}\left(t\right)\right]+\frac{\partial V_{H1}}{\partial x_{H1}} \\ \left[-\lambda_{1}\left(\frac{c_{I}-\alpha_{1}}{2\lambda_{1}}\right)^{2}\left(\frac{\partial V_{H1}}{\partial x_{H1}}\right)^{-2}+\ln\left(1+\beta+\beta_{H}\right)\right. \\ \left. \frac{1}{c_{M}+c_{H}}\left[b+\frac{\partial V_{H}}{\partial x_{H}}\ln\left(1+\beta+\beta_{H}\right)\right]-\delta x_{H1}\left(t\right)\right] \end{matrix}$ (53)

$\rho V_{H2}=\left[-\frac{c_{F}+c_{H}}{2}\left(\frac{\lambda_{2}+\lambda_{H}}{c_{F}+c_{H}}\frac{\partial V_{H2}}{\partial x_{H2}}\right)^{2}\right.\left.+l_{2}x_{H2}\left(t\right)\right]+\frac{\partial V_{H2}}{\partial x_{H2}}\left[\frac{\left(\lambda_{2}+\lambda_{H}\right)^{2}}{c_{F}+c_{H}}\frac{\partial V_{H2}}{\partial x_{H2}}-\delta x_{H2}\left(t\right)\right]$ (54)

Let $V_{H1}^{*}=m_{5}x_{H1}+m_{6}$,$V_{H2}^{\text{*}}=m_{7}x_{H2}+m_{8}$, wherein, *m*_5_, *m*_6_, *m*_7_ and *m*_8_ are all constants. The parameters of the optimal social welfare function can be obtained by calculation as follows:

$\left\{\begin{matrix} m_{5}=\frac{l_{1}}{\rho+\delta} \\ m_{6}=\frac{1}{\rho}\left[b\frac{1}{c_{M}+c_{H}}\left[b+\frac{l_{1}}{\rho+\delta}\ln\left(1+\beta+\beta_{H}\right)\right]+\frac{\left(c_{I}-\alpha_{1}\right)^{2}}{2\lambda_{1}}\left(\frac{l_{1}}{\rho+\delta}\right)^{-1}\right. \\ -\left.\frac{1}{2}\frac{1}{c_{M}+c_{H}}\left[b+\frac{l_{1}}{\rho+\delta}\ln\left(1+\beta+\beta_{H}\right)\right]^{2}\right]+\frac{l_{1}}{\rho+\delta}\frac{1}{\rho}\ln\left(1+\beta+\beta_{H}\right) \\ \left[b+\frac{l_{1}}{\rho+\delta}\ln\left(1+\beta+\beta_{H}\right)\right]\frac{1}{c_{M}+c_{H}}-\frac{l_{1}}{\rho+\delta}\frac{1}{\rho}\lambda_{1}\left(\frac{c_{I}-\alpha_{1}}{2\lambda_{1}}\right)^{2}\left(\frac{l_{1}}{\rho+\delta}\right)^{-2} \end{matrix}\right.$ (55)

$\left\{\begin{matrix} m_{7}=\frac{l_{2}}{\rho+\delta} \\ m_{8}=-\frac{1}{\rho}\frac{c_{F}+c_{H}}{2}\left(\frac{\lambda_{2}+\lambda_{H}}{c_{F}+c_{H}}\frac{l_{2}}{\rho+\delta}\right)^{2}+\frac{l_{2}}{\rho+\delta}\frac{1}{\rho}\frac{\left(\lambda_{2}+\lambda_{H}\right)^{2}}{c_{F}+c_{H}}\frac{l_{2}}{\rho+\delta} \end{matrix}\right.$ (56)

Therefore, it can be concluded that:

$\begin{matrix} V_{H1}^{*}=\frac{l_{1}}{\rho+\delta}x_{H1}+\frac{1}{\rho}\left[b\frac{1}{c_{M}+c_{H}}\left[b+\frac{l_{1}}{\rho+\delta}\ln\left(1+\beta+\beta_{H}\right)\right]+\frac{\left(c_{I}-\alpha_{1}\right)^{2}}{2\lambda_{1}}\left(\frac{l_{1}}{\rho+\delta}\right)^{-1}-\right. \\ \left.\frac{1}{2}\frac{1}{c_{M}+c_{H}}\left[b+\frac{l_{1}}{\rho+\delta}\ln\left(1+\beta+\beta_{H}\right)\right]^{2}\right]+\frac{l_{1}}{\rho+\delta}\frac{1}{\rho}\ln\left(1+\beta+\beta_{H}\right)\frac{1}{c_{M}+c_{H}} \\ \left[b+\frac{l_{1}}{\rho+\delta}\ln\left(1+\beta+\beta_{H}\right)\right]-\frac{l_{1}}{\rho+\delta}\frac{1}{\rho}\lambda_{1}\left(\frac{c_{I}-\alpha_{1}}{2\lambda_{1}}\right)^{2}\left(\frac{l_{1}}{\rho+\delta}\right)^{-2} \end{matrix}$ (57)

$V_{H2}^{\text{*}}=\frac{l_{2}}{\rho+\delta}x_{H2}-\frac{1}{\rho}\frac{c_{F}+c_{H}}{2}\left(\frac{\lambda_{2}+\lambda_{H}}{c_{F}+c_{H}}\frac{l_{2}}{\rho+\delta}\right)^{2}+\frac{l_{2}}{\rho+\delta}\frac{1}{\rho}\frac{\left(\lambda_{2}+\lambda_{H}\right)^{2}}{c_{F}+c_{H}}\frac{l_{2}}{\rho+\delta}$ (58)

In this case,

$M_{H1}^{\text{*}}\left(t\right)\text{=}\frac{1}{c_{M}+c_{H}}\left[b+\frac{l_{1}}{\rho+\delta}\ln\left(1+\beta+\beta_{H}\right)\right]$ ,$I_{H1}^{\text{*}}\left(t\right)\text{=}-\frac{c_{I}-\alpha_{1}}{2\lambda_{1}}\left(\frac{l_{1}}{\rho+\delta}\right)^{-1}$ (59)

$F_{H2}^{\text{*}}\left(t\right)\text{=}\frac{\lambda_{2}+\lambda_{H}}{c_{F}+c_{H}}\frac{l_{2}}{\rho+\delta}$ (60)

**Appendix 3**

Take the derivatives of *M_G_*_1_ and *I_G_*_1_ respectively with respect to (17), and take the derivatives of *F_G_*_2_ with respect to (18), and set them equal to zero, we can get:

$M_{G1}^{\text{*}}\left(t\right)\text{=}\frac{1}{c_{M}+c_{G}}\left[b+b_{G}+\frac{\partial V_{H1}}{\partial x_{H1}}\ln\left(1+\beta+\beta_{G}\right)\right]$,$I_{G1}^{\text{*}}\left(t\right)\text{=}-\frac{c_{I}}{2\lambda_{1}}\left(\frac{\partial V_{G1}}{\partial x_{G1}}\right)^{-1}$ (61)

$F_{G2}^{\text{*}}\left(t\right)\text{=}\frac{\lambda_{2}+\lambda_{G}}{c_{F}+c_{G}}\frac{\partial V_{G2}}{\partial x_{G2}}$ (62)

Substituting (61) into (17) and substituting (62) into (18), we can get:

$\begin{matrix} \rho V_{G1}=\left[\left(b+b_{G}\right)\frac{1}{c_{M}+c_{G}}\left[b+b_{G}+\frac{\partial V_{H1}}{\partial x_{H1}}\ln\left(1+\beta+\beta_{G}\right)\right]-c_{I}I_{G1}\left(t\right)-\frac{1}{2}\frac{1}{c_{M}+c_{G}}\right. \\ \left.\left[b+b_{G}+\frac{\partial V_{H1}}{\partial x_{H1}}\ln\left(1+\beta+\beta_{G}\right)\right]^{2}+l_{1}x_{G1}\left(t\right)\right]+\frac{\partial V_{G1}}{\partial x_{G1}}\left[-\lambda_{1}\left(\frac{c_{I}}{2\lambda_{1}}\right)^{2}\left(\frac{\partial V_{G1}}{\partial x_{G1}}\right)^{-2}-\delta x_{G1}\left(t\right)\right] \\ +\frac{\partial V_{G1}}{\partial x_{G1}}\ln\left(1+\beta+\beta_{G}\right)\frac{1}{c_{M}+c_{G}}\left[b+b_{G}+\frac{\partial V_{H1}}{\partial x_{H1}}\ln\left(1+\beta+\beta_{G}\right)\right] \end{matrix}$(63)$\rho V_{G2}=\left[-\frac{c_{F}+c_{G}}{2}\left(\frac{\lambda_{2}+\lambda_{G}}{c_{F}+c_{G}}\frac{\partial V_{G2}}{\partial x_{G2}}\right)^{2}\right.\left.+l_{2}x_{G2}\left(t\right)\right]+\frac{\partial V_{G2}}{\partial x_{G2}}\left[\left(\lambda_{2}+\lambda_{G}\right)\frac{\lambda_{2}+\lambda_{G}}{c_{F}+c_{G}}\frac{\partial V_{G2}}{\partial x_{G2}}-\delta x_{G2}\left(t\right)\right]$(64)

Let $V_{G1}^{*}=m_{9}x_{G1}+m_{10}$,$V_{G2}^{\text{*}}=m_{11}x_{H2}+m_{12}$, wherein, *m*_9_, *m*_10_, *m*_11_ and *m*_12_ are all constants. The parameters of the optimal social welfare function can be obtained by calculation as follows:

$\left\{\begin{matrix} m_{9}=\frac{l_{1}}{\rho+\delta} \\ m_{10}=\frac{1}{\rho}\left[\left(b+b_{G}\right)\frac{1}{c_{M}+c_{G}}\left[b+b_{G}+\frac{l_{1}}{\rho+\delta}\ln\left(1+\beta+\beta_{G}\right)\right]-\frac{1}{2}\frac{1}{c_{M}+c_{G}}\right. \\ \left.\left[b+b_{G}+\frac{l_{1}}{\rho+\delta}\ln\left(1+\beta+\beta_{G}\right)\right]^{2}\right]-\frac{1}{\rho}c_{I}I_{G1}\left(t\right)+\frac{l_{1}}{\rho+\delta}\frac{1}{\rho}\ln\left(1+\beta+\beta_{G}\right)\frac{1}{c_{M}+c_{G}} \\ \left[b+b_{G}+\frac{l_{1}}{\rho+\delta}\ln\left(1+\beta+\beta_{G}\right)\right]-\frac{l_{1}}{\rho+\delta}\frac{1}{\rho}\lambda_{1}\left(\frac{c_{I}}{2\lambda_{1}}\right)^{2}\left(\frac{l_{1}}{\rho+\delta}\right)^{-2} \end{matrix}\right.$(65)$\left\{\begin{matrix} m_{11}=\frac{l_{2}}{\rho+\delta} \\ m_{12}=-\frac{1}{\rho}\frac{c_{F}+c_{G}}{2}\left(\frac{\lambda_{2}+\lambda_{G}}{c_{F}+c_{G}}\frac{l_{2}}{\rho+\delta}\right)^{2}+\frac{l_{2}}{\rho+\delta}\frac{1}{\rho}\left(\lambda_{2}+\lambda_{G}\right)\frac{\lambda_{2}+\lambda_{G}}{c_{F}+c_{G}}\frac{l_{2}}{\rho+\delta} \end{matrix}\right.$ (66)

Therefore, it can be concluded that:

$\begin{matrix} V_{G1}^{*}=\frac{l_{1}}{\rho+\delta}x_{G1}+\frac{1}{\rho}\left[\left(b+b_{G}\right)\frac{1}{c_{M}+c_{G}}\left[b+b_{G}+\frac{l_{1}}{\rho+\delta}\ln\left(1+\beta+\beta_{G}\right)\right]-c_{I}I_{G1}\left(t\right)-\frac{1}{2}\frac{1}{c_{M}+c_{G}}\right. \\ \left.\left[b+b_{G}+\frac{l_{1}}{\rho+\delta}\ln\left(1+\beta+\beta_{G}\right)\right]^{2}\right]+\frac{l_{1}}{\rho+\delta}\frac{1}{\rho}\ln\left(1+\beta+\beta_{G}\right)\frac{1}{c_{M}+c_{G}} \\ \left[b+b_{G}+\frac{l_{1}}{\rho+\delta}\ln\left(1+\beta+\beta_{G}\right)\right]-\frac{l_{1}}{\rho+\delta}\frac{1}{\rho}\lambda_{1}\left(\frac{c_{I}}{2\lambda_{1}}\right)^{2}\left(\frac{l_{1}}{\rho+\delta}\right)^{-2} \end{matrix}$(67)$V_{G2}^{\text{*}}=\frac{l_{2}}{\rho+\delta}x_{G2}-\frac{1}{\rho}\frac{c_{F}+c_{G}}{2}\left(\frac{\lambda_{2}+\lambda_{G}}{c_{F}+c_{G}}\frac{l_{2}}{\rho+\delta}\right)^{2}+\frac{l_{2}}{\rho+\delta}\frac{1}{\rho}\left(\lambda_{2}+\lambda_{G}\right)\frac{\lambda_{2}+\lambda_{G}}{c_{F}+c_{G}}\frac{l_{2}}{\rho+\delta}$ (68)

In this case,

$M_{G1}^{\text{*}}\left(t\right)\text{=}\frac{1}{c_{M}+c_{G}}\left[b+b_{G}+\frac{l_{1}}{\rho+\delta}\ln\left(1+\beta+\beta_{G}\right)\right]$,$I_{G1}^{\text{*}}\left(t\right)\text{=}-\frac{c_{I}}{2\lambda_{1}}\left(\frac{l_{1}}{\rho+\delta}\right)^{-1}$ (69)

$F_{G2}^{\text{*}}\left(t\right)\text{=}\frac{\lambda_{2}+\lambda_{G}}{c_{F}+c_{G}}\frac{l_{2}}{\rho+\delta}$ (70)
