# Supplementary material for: Health improvement of the elderly in five Central Asian countries during COVID-19 based on difference game
Source: PLoS One. 2023 Dec 5;18(12):e0294697. doi: 10.1371/journal.pone.0294697 (PMC10697614; doi:10.1371/journal.pone.0294697)
Supplement: Attachment — Submitted filename: Response to reviewer2.docx [file pone.0294697.s002.docx]

Dear Editors and Reviewers:

# Many thanks for your valuable comments and suggestions on our manuscript entitled “Health improvement of the elderly in five Central Asian countries during COVID-19 based on difference game” (Manuscript ID: PONE-D-23-25723). The comments and suggestions are very helpful for improving our paper. We have made revision based on the comments and suggestions. Please find our response as follows, and we have made revision which marked in blue in the paper. Attached please find the revised version, which we would like to submit for your kind consideration.

**Point ：**

The statements seem to provide a foundation for research hypotheses related to COVID-19 management in Central Asian countries, particularly concerning the elderly population and healthcare infrastructure. To formulate specific hypotheses, you would need to state the research questions you want to address and the relationships you wish to investigate. Additionally, these hypotheses should be framed more precisely, with clear independent and dependent variables, to make them suitable for empirical testing.In general, the conclusions appear to be logically derived from the information presented in the text, with a focus on the dynamic nature of healthcare resource allocation during the pandemic.

**Response :**

Thank you very much for your suggestion. In the revised version, in order to form specific hypotheses, this paper states the research problem that is intended to be solved. For details, see lines 226-231 in blue. In a modified version, this article states the relationship you want to investigate in order to form a specific hypothesis. For details, see lines 208-225 in blue.

In the revised version, in order that the framework of these assumptions should be more precise, the independent and dependent variables are stated in the assumptions. For details, see lines 239-240, 249-250, 253-255 and 257-258 in blue.

In the revised version, this paper describes the dynamic nature of health care resource allocation during a pandemic. For details, see lines 138-168 in blue.
